# *Symphytum officinale* L. Plays a Dominant Role in Mitigating Nitrogen Accumulation in Soil Under Long-Term Irrigation with Treated Poultry Wastewater

**DOI:** 10.3390/plants15030433

**Published:** 2026-01-30

**Authors:** Jiaxin Li, Ruilun Zheng, Chuansheng Chen, Peixin Wang, Xinjie Yang, Zhicheng Yang, Qinghai Wang

**Affiliations:** 1 Institute of Grassland, Flowers and Ecology, Beijing Academy of Agriculture and Forestry Sciences, Beijing 100097, China; 13871391139@163.com (J.L.); zhengruilun@baafs.net.cn (R.Z.) wpxin2636@163.com (P.W.); yangxinjie123789@163.com (X.Y.); 2 College of Environmental Science and Engineering, Central South University of Forestry and Technology, Changsha 410004, China; t19991064@csuft.edu.cn

**Keywords:** treated poultry wastewater, *Symphytum officinale*, soil property, soil bacterial community

## Abstract

Comfrey (*Symphytum officinale* L.) was a promising crop in the integrated crop–livestock pattern. However, the impact of long-term irrigation with treated poultry wastewater (TPW) on soil chemical properties and bacterial community, as well as the contribution of comfrey to mitigate N accumulation in soil, remain unclear. This study investigated the changes in chemical and microbiological characteristics of the comfrey soil under six-year TPW irrigation (grassland) in comparison with the adjacent conventional crop soil irrigated with freshwater (farmland). Results showed that N accumulation in comfrey accounted for 66.0% of the N input from TPW irrigation. In grassland, soil pH at all depths increased by one unit and EC in the subsoil increased by 33.5–42.4%, while TN and NO_3_^−^-N in the surface soil decreased by 26.7% and 64.5%, respectively. The composition and structure of the bacterial community in the grassland dramatically changed, and the relative abundances of nitrite-oxidizing bacteria *Nitrospira* and ammonifying bacterium *Flavobacterium* and *Pseudomonas* increased by 0.1–3.6-, 3.8–11.0- and 0.1–6.0-fold, respectively, while those of saline-alkali-sensitive bacteria *Sphingomonas* decreased by 72.3–83.2% in the subsoil. Soil pH and NO_3_^−^-N were the primary factors influencing changes in bacterial communities. These findings revealed that there was no nitrogen accumulation, but alkalization occurred in the comfrey field under long-term TPW irrigation, which highlighted the prospective application of comfrey in the crop–livestock system.

## 1. Introduction

The continuous increase in the number of livestock and poultry has made tremendous contributions in providing food security to the growing global population; however, it has inevitably induced numerous environmental pollution and public health issues due to the generation and discharge of substantial quantities of waste products [1,2]. Besides solid waste, inadequately treated wastewater poses serious pollution threats to environments and has received a great deal of attention worldwide [3]. The negative environmental impacts of wastewater reuse for agricultural irrigation mainly included contamination of farmland, crops and groundwater, as well as the alteration in physicochemical properties and microbiota of soil; and these potential risks primarily arose from salts, microbial pathogens, heavy metals, potentially toxic elements, contaminants of emerging concern (such as pharmaceuticals), antibiotic resistance genes and disinfection by-products introduced by reclaimed wastewater [4,5]. Therefore, the adequate treatment and subsequent disposal measures for wastewater were paramount to the sustainable development of intensive poultry farming operations. The land disposal of poultry wastewater for irrigation purposes following suitable treatment was a popular approach due to the enormous number of organic materials and essential nutrients in the wastewater [1]. Treated poultry wastewater (TPW) reuse for crop irrigation not only solved environmental pollution issues caused by its improper discharge, but also served as an effective measure to alleviate the pressure of agricultural water resource scarcity. However, despite these benefits of irrigation with treated wastewater, its drawbacks should not be ignored, e.g., the high concentration of dissolved ions leading to elevated salinity [6] and an increase in soil electrical conductivity [7], high levels of nutrients causing nitrogen (N) accumulation in the soils and elevating the risk of nutrition loss through surface runoff [3,8], especially nitrate (NO_3_^−^), posing significant environmental and human health risks [9]. In this context, it was essential to carefully consider the physiochemical properties of the soil when irrigated with treated wastewater [6].

In real applications, to meet the demand for the disposal of continuously generated wastewater, the agricultural land receiving wastewater had to be irrigated with high frequency, while the conventional crops, such as summer corn, could not normally grow and develop owing to their inability to adapt to the long-term excessive input of water and nutrients from the frequent irrigation [10]. Great efforts have been made to find alternative plants with economic value and high tolerance to frequent TPW irrigation-related stress. Comfrey (*Symphytum officinale* L.) was a valuable forage due to its rapid growth, high yields and protein contents and ease of management [11]. In addition, the plant had high tolerance to waterlogging conditions and the ability for efficient uptake and translocation of nutrient elements. These beneficial characteristics qualified it as a suitable crop candidate in the integrated crop–livestock system. However, little information is available about whether long-term TPW irrigation could cause N accumulation in soils and about the contribution of the plant to the mitigation of N load in soil. On the other side, soil microbiome played an important role in maintaining ecosystem stability, productivity and resilience towards stress and disturbance [12], but its composition and diversity usually exhibited pronounced vulnerability to frequent anthropogenic disturbances, including wastewater irrigation [7]. However, the microbial responses to the switch from conventional crops to comfrey and simultaneous TPW irrigation remained relatively unexplored.

Hence, the objectives of this study were to achieve the following: (1) investigate variations in soil chemical properties in the comfrey field relative to the adjacent conventional farmland, determine the extent of N accumulation in soil and evaluate the contribution of comfrey to reducing N accumulation; and (2) reveal the response characteristics of bacteria and determine the main factors driving the shifts in soil bacterial communities correlated with soil properties.

## 2. Results and Discussion

### 2.1. Contribution of S. Officinale to Removing N from TPW-Receiving Soil

Under TPW irrigation, *S. officinale* exhibited rapid growth and development, and its total yield (dry weight) reached 11.7 t ha^−1^ for the four cuttings (Table 1), suggesting its strong adaptability to TPW. A previous study confirmed that the treated wastewater was more suitable for grassland than shrubland [13]. The yield of each cutting firstly increased and then decreased with cutting date, peaking at the second mowing (3.8 t ha^−1^); and the yield at the fourth cutting (1.7 t ha^−1^) was significantly lower than the first three mowings. The N content in the aboveground parts of *S. officinale* displayed a pattern of the succeeding cuttings being higher than preceding ones, with the fourth cutting significantly exceeding the first three cuttings. The timely mowing was one of the most important means to improve the production and quality of forages. The four-time cutting and each cutting performed after the initial flowering stage were recommended for the management of S. officinale in northern China and similar regions. Previous studies have confirmed that comfrey was a valuable forage. Nevertheless, TPW irrigation might have impact on its quality, which should be included in subsequent studies.

Based on the irrigation amount and the N content of wastewater, the TPW irrigation-derived N input to the grassland was determined to be 514.7 kg ha^−1^, which was obviously higher than N input in the adjacent conventional farmlands. Based on the yield and N content in the aboveground biomass of *S. officinale*, the annual N accumulation of the grass was 339.4 kg ha^−1^, accounting for a major fraction (66.0%) of the N input from wastewater irrigation. The threshold value of TN content in reclaimed water reuse for agricultural irrigation was not imposed by the regulation (EU) 2020/741 on minimum requirements for agricultural water reuse, but the Italian regulation regulated it, considering a limit of 15.0 mg L^−1^ [14]. Accordingly, high nitrogen content was one of the most prominent characteristics of TPW used in this study (Table 2). The information further demonstrated that *S. officinale* had a remarkable ability to capture N from the soil, playing a dominant role in reducing N accumulation in the grassland soil caused by TPW irrigation. In the present study, plant samples were collected only in 2023; the next research work should collect plant samples over multiple consecutive years to exclude the influence of interannual meteorological conditions, enabling a more accurate analysis of the N accumulation capacity of the plant.

### 2.2. Variations in Soil Chemical Properties in Grassland vs. Farmland Under Long-Term TPW Irrigation

**Figure 1:**
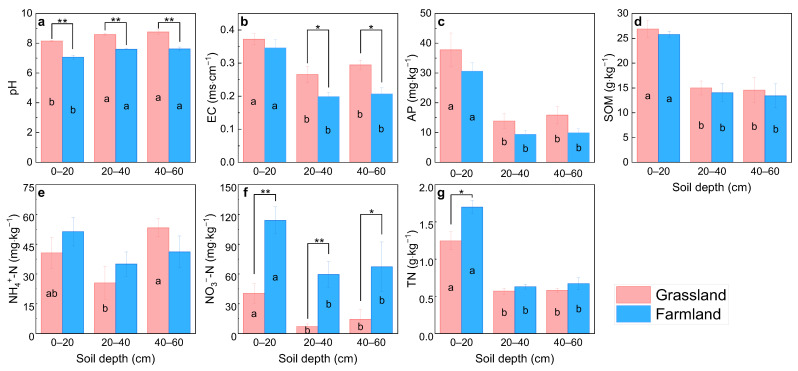
Basic soil properties of grassland and adjacent farmland at different depths: (**a**) pH; (**b**) EC; (**c**) AP; (**d**) SOM; (**e**) NH_4_^+^-N; (**f**) NO_3_^−^-N; (**g**) TN. All data are expressed as means ± SE. Different letters indicate significant differences between different soil depths according to Duncan’s test (*p* < 0.05). * and ** indicate significant differences between grassland and farmland at *p* < 0.05 and *p* < 0.01, respectively.

The pH levels of the three soil layers (0–20, 20–40 and 40–60 cm) in the grassland irrigated with TPW ranged from 8.1 to 8.8, which was significantly higher than the 7.1 to 7.6 found in the farmland (Figure 1a). The variation in soil pH with depth followed the same pattern for both grassland and farmland soils, being significantly higher in the deeper soil layers than the surface layer (0–20 cm), while no significant difference was observed between layers of 20–40 and 40–60 cm. This indicated that long-term TPW irrigation can lead to soil alkalization, which may be due to the accumulation of basic cations often present in treated wastewater [15]. Electrical conductivity (EC) in the surface soil showed no significant difference between grassland and farmland, whereas EC in deeper soil layers was significantly higher in grassland than in farmland (Figure 1b), which closely resembles findings from an earlier study, that the increase in EC in the lower soil horizons was the main effect of treated wastewater irrigation due to the downward flushing of dissolved salts by irrigation [16]. This indicated that long-term TPW irrigation tended to lead to soil salinization, which should be given more prominence in the long-standing practice of wastewater irrigation. The secondary soil salinization was one of the most significant problems faced in wastewater reuse for agricultural irrigation [17], which had negative effects on soil quality and crop growth. The vigorous growth of *S. officinale* indicated its tolerance to a certain degree of soil salinization. Plants mitigated salt stress by reducing water loss while maximizing water uptake, and also minimized the harmful effects of ionic stress by the exclusion of ions from leaf tissues and by compartmentalization into vacuoles [18]. Additionally, the synergistic effect of root exudates and rhizosphere microorganisms played an important role in plant adaptation to saline soils [19]. The main tolerance mechanisms of *S. officinale* to salt stress should be further explored in future work to help the plant function more effectively in the crop–livestock system. To mitigate potential risks of TRW irrigation, establishing and implementing strict quality standards for wastewater reuse was an essential safeguard. The European Union has approved the Regulation (EU) 2020/741 on minimum requirements for agricultural water reuse with the aim of ensuring its safe application in agriculture [20,21]. In addition, predicting the change and the development of soil salinization was critical for sustainable TPW irrigation management, and much effort has been made to develop various models to simulate salinity dynamics in soil [22]. In order to accurately assess the sustainability of the integrated crop–livestock pattern, future work should predict the time required to reach the critical level of soil salinization, which would ultimately prevent further cultivation of comfrey under long-term TPW irrigation. Grassland showed slightly higher available phosphorus (AP) and soil organic matter (SOM) than farmland, while the difference was not significant. For both grassland and farmland, AP and SOC varied significantly in soil depth, with the surface soil being higher than subsoils (Figure 1c,d). No significant differences were found in NH_4_^+^-N contents between grassland and farmland (Figure 1e). The contents of NO_3_^−^-N and total nitrogen (TN) were lower in grassland than in farmland across soil depths, while TN contents at the depths of 20–40 and 40–60 cm were not significant (*p* > 0.05) (Figure 1f,g). These results indicated that TPW irrigation did not cause N accumulation in the soil-planted *S. officinale* relative to adjacent conventional farmland. In addition to being rich in high levels of nutrients (N and P), the poultry wastewater often contained a large amount of dissolved organics, some toxic substances and harmful microorganisms; heavy metals, antibiotics and pathogens, especially, can accumulate in soil, posing a potential threat to human and environmental health [23,24,25]. The present study only focused on N cycling, without addressing the transformation and migration of other substances, as well as pathogens. These important questions should be a major concern which needs to be addressed in future research.

### 2.3. Effects of TPW Irrigation on Bacterial Community

#### 2.3.1. Soil Bacterial Diversity

**Figure 2:**
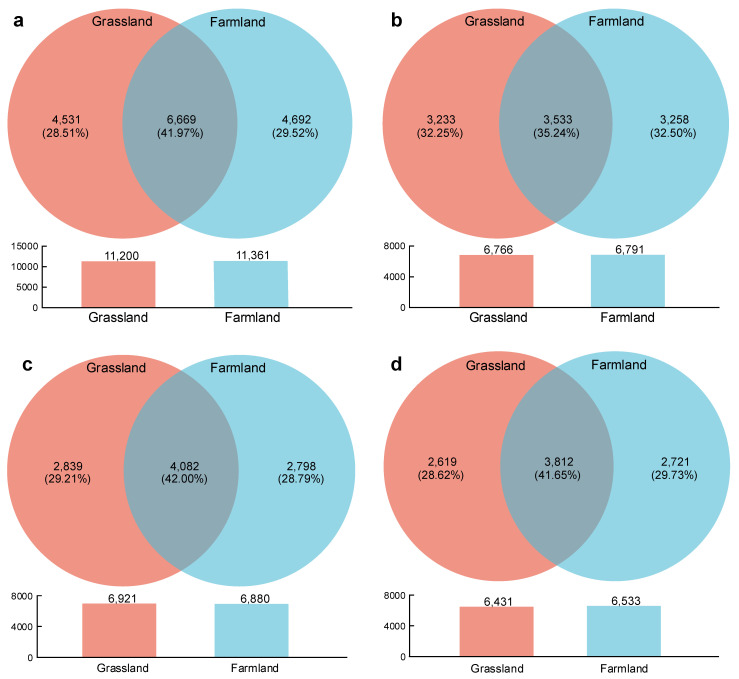
Venn diagram of bacterial OTU numbers of grassland and adjacent farmland at different depths: (**a**) total, (**b**) 0–20 cm, (**c**) 20–40 cm and (**d**) 40–60 cm.

High-throughput sequencing analysis displayed that a total of 15892 bacterial OTUs were detected in all soil samples, and the OTU number in farmland was slightly higher than that in grassland (Figure 2a). Bacterial OTU analysis revealed that 3533, 4082 and 3812 OTUs were common to soil samples at 0–20, 20–40 and 40–60 cm depths in grassland and farmland, respectively, and the percentage of shared OTUs in topsoil (35.24%) was the lowest among soils at all depths (Figure 2b–d). Consequently, the percentage of unique OTUs showed the opposite pattern and was higher in the 0–20 cm layer than in other soil horizons, indicating stronger responses of bacterial community in the topsoil to TPW irrigation. The high N inputs by irrigation, as evidenced by the high N content in wastewater, could be an important reason for the differentiation of bacterial community composition in different soil layers, because microbes at the surface soil layers were generally more sensitive to N additions than those at deeper soil layers [26].

**Figure 3:**
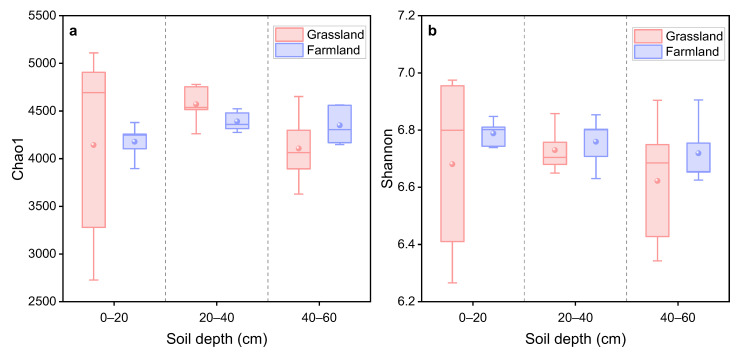
Alpha diversity of bacteria in grassland and adjacent farmland at different depths: (**a**) Chao1 index and (**b**) Shannon index. All data are expressed as means ± SE.

Alpha diversity, reflecting bacterial community richness and diversity, was evaluated using the Chao1 and Shannon indices. As shown in Figure 3, the Chao1 and Shannon indices in the grassland were lower than those in the farmland (with the exception of Chao1 in the 20–40 cm depth), but the difference was not statistically significant. In addition, there is no significant difference in the alpha diversity between different soil depths. The result indicated that, within six years after the conversion of farmland to grassland irrigated with TPW, species richness and evenness of bacterial communities did not significantly change, but showed a certain downward trend. This declining tendency may be related to the inputs of large amounts of nutrients from TPW irrigation. The previous study has demonstrated that high nutrient concentrations caused more negative interactions between bacterial species, resulting in a loss of biodiversity and a decrease in the microbial community stability [27].

**Figure 4:**
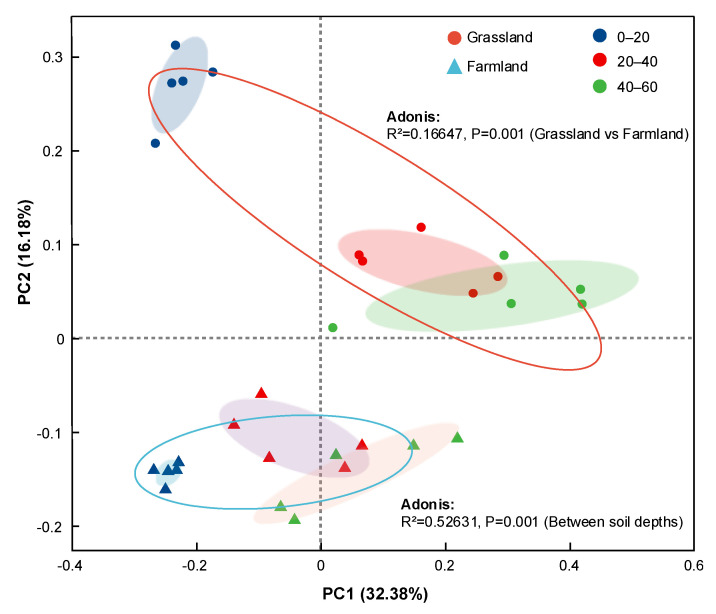
PCoA of bacterial communities in grassland and adjacent farmland at different depths on the basis of Bray-Curtis distance.

PCoA based on Bray–Curtis dissimilarity at the OTU level revealed distinct clustering patterns between grassland and farmland, as well as among different soil depths (Figure 4), with PC1 and PC2 contributing 32.38% and 16.18% of the total variation, respectively. The grassland group showed a clear separation from the farmland group, indicating a shift in bacterial structure induced by TPW irrigation. The different soil depths also displayed a clear separation from each other, with partial overlap between 20 and 40 with the 40–60 group. The Adonis test further displayed that irrigation with wastewater and soil depth showed a significant effect on bacterial composition at the OTU level (R^2^ = 0.1665, *p* = 0.001; R^2^ = 0.5263, *p* = 0.001).

#### 2.3.2. Bacterial Community Composition

**Figure 5:**
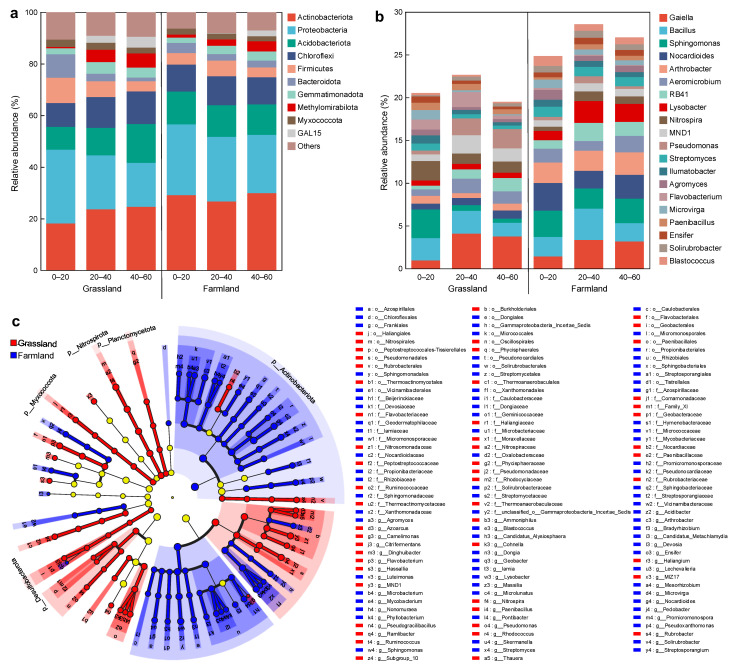
Bacterial relative abundances at (**a**) phylum and (**b**) genus levels; (**c**) LefSe analysis (LDA = 3.5, circles represent phylogenetic levels from phylum to genus, and the diameter of each circle is proportional to the abundance of the group).

At the phylum level, the dominant bacterial phyla in both grassland and farmland included Actinobacteria, Proteobacteria, Acidobacterioa, Chioroflexi, Firmicute and Bacteroidota, collectively constituting 74.7–88.1% of the bacteria (Figure 5a). For farmland, no significant difference existed in the abundance of Actinobacteria, Proteobacteria and Acidobacterioa among different soil depths (*p* = 0.4564, *p* = 0.3863, *p* = 0.9741, respectively). However, the relative abundance of the dominant bacterial phyla in the grassland was significantly affected by soil depth (*p* < 0.05). The abundance of Actinobacteria, Acidobacterioa and Methylomirabilota increased significantly with soil depth, while the abundance of Proteobacteria, Firmicute and Bacteroidota decreased significantly with soil depth. These results suggested that the interaction between TPW irrigation and soil depth significantly influenced bacterial composition. Members of the phylum Actinobacteria had a crucial role in organic matter turnover and the breakdown of recalcitrant molecules, manifesting their potential importance in the terrestrial carbon cycle and improving soil health [28,29]. Compared to farmland, the abundance of Actinobacteria in grassland remarkedly decreased in surface soils but not in deeper soils. A similar phenomenon was also observed in a study on the response of soil microbes to long-term N additions, suggesting that microorganisms can adjust their community structure and survival strategies to acclimate to changes in soil nutrient availability caused by N addition [26]. Major processes of the N cycle mainly included ammonification, nitrification, denitrification and anammox [30]. The Proteobacteria comprised many N-fixing, ammonia-oxidizing and denitrifying taxa, and played pivotal roles in soil N cycling [31]. The more abundant Proteobacteria in the surface soil indicated the higher denitrification efficiency in this soil layer [32]. In the 0–20 cm soil layers, the abundance of Bacteroidota and Firmicutes was significantly higher in grassland than in farmland, while the abundance of Methylomirabilota was significantly lower in grassland than in farmland. Bacteroidota played an important role in N cycling and energy conversion in ecosystems, as well as in the decomposition of macromolecular organic matter. Firmicutes was involved in the denitrification and had acidification effects on agricultural waste [33]. Therefore, the enrichment of Firmicutes in topsoil in grassland could improve soil environment with high N and salinity caused by irrigation.

The top 20 identified genera in the soil of grassland and adjacent farmland are shown in Figure 5b. Among these, the top 10 genera with the highest relative abundance included *Gaiella*, *Bacillus*, *Sphingomonas*, *Nocardioides*, *Arthrobacter*, *Aeromicrobium*, RB41, *Lysobacter*, *Nitrospira* and MND1, and half of them belonged to *Actinobacteriota*. Compared with the farmland, the relative abundance of *Nitrospira*, a globally distributed group of nitrite oxidizers with good adaptation to a wide range of environments [34,35], was significantly increased, particularly in the topsoil of grassland. Notably, this increased abundance of *Nitrospira* may be attributed to the high content nitrite in the grassland soil, conferring a competitive advantage in the soil microbial ecosystem by facilitating the complete nitrification of ammonia input by irrigation. A recent study also confirmed that *Nitrospira* was one of the key N-cycling microbes in the soil under long-term irrigation with municipally treated wastewater [9]. The LEfSe analysis showed that *Nitrospira* (LDA = 4.01) was significantly enriched in the grassland, revealing that it was a key differential taxon in the grassland (Figure 5c). In the present study, the majority of N in TPW existed as organic forms, while the inorganic N (mainly in the form of ammonium-N) only accounted for less than 15% (Table 2). The transformation of organic N to inorganic N was the key step for decreasing N accumulation in soil by plants. This transformation was primarily driven by microbes [36]; *Pseudomonas*, *Flavobacterium* and *Bacillus* were important drivers [37,38]. The relative abundance of *Flavobacterium* in the surface soil (0–20 cm) and those of *Pseudomonas* at the deeper layers (20–40 and 40–60 cm) was significantly higher in grassland than in farmland, suggesting that they played a principal role in mineralizing organic N from TPW to ammonium, which was subsequently taken up by plants. In addition to bacteria, fungi, invertebrates and plants also played a crucial role in the N cycle [39]. In terms of plants, they not only directly influenced the N cycle via the uptake of available N, but plant-mediated shifts in soil microbes could also affect the N cycle. However, these important influencing factors have not been considered in this study. It is therefore incumbent on future work to focus on the effects of multifactorial interactions on the N cycle in soil irrigated by TPW and the underlying mechanisms. The relative abundance of *Nocardioides* and *Arthrobacter* was significantly reduced in the grassland. These two genera were well known for their roles in metabolizing s-triazine herbicides via an initial hydrolytic displacement of chloride [40], and were the dominant component of atrazine degraders in the maize rhizosphere [41]. The decrease in their relative abundance might be related to the shift in planting patterns from wheat–corn rotation to forage crops. In this study, herbicides, such as atrazine, were no longer applied after switching to forage cultivation, and thus herbicide residue in soil decreased, which resulted in the reduction in bacteria capable of degrading these herbicides. *Sphingomonas* and *Lysobacter*, the core bacterial genera in wheat field [42], were also reduced, particularly in the soil of 20–40 and 40–60 cm depths. This aligns with a prior study, confirming that irrigation with reclaimed water increased soil pH and reduced the relative abundance of *Sphingomonas* [43]. *Sphingomonas* was sensitive to saline–alkaline stress, and could be replaced by halophilic species in the soil under elevated salinity; its population density decreased with the increase in soil EC and pH [44,45]. Therefore, the reduction in its abundance in grassland might be related to the significant rise in soil EC and pH resulting from the shift in planting patterns. On the other hand, *Sphingomonas* alleviated salinity stress and significantly improved the salt tolerance of the plant [46]; hence, its significantly higher abundance in the topsoil in the present study played an important role in promoting the growth of *S. officinale* under salinity stress from irrigation.

#### 2.3.3. Relation Between Bacterial Communities and Soil Properties

**Figure 6:**
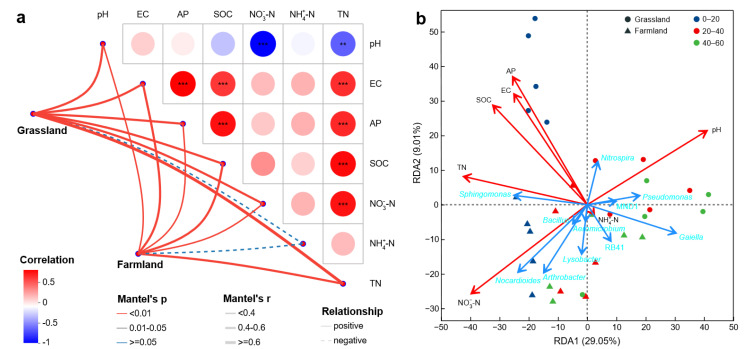
Relationship between bacteria and soil environmental factors of grassland and adjacent farmland based on (**a**) Mantel test and (**b**) RDA. ** *p* < 0.01, *** *p* < 0.001.

Mantel test analysis revealed a significant positive correlation between the overall bacterial community and all soil properties examined, except for NH_4_^+^-N (Figure 6a). In grassland, TN had the greatest contributions to the composition and structure of bacterial communities (Mantel’s r = 0.7044, *p* = 0.001), followed by AP (Mantel’s r = 0.5267, *p* = 0.001) and pH (Mantel’s r = 0.5202, *p* = 0.003). In farmland, SOC exhibited the strongest influence on the bacterial community (Mantel’s r = 0.5406, *p* = 0.001), followed by TN (Mantel’s r = 0.5220, *p* = 0.001) and EC (Mantel’s r = 0.4665, *p* = 0.001). This result suggested that TN, AP and pH were important in the grassland, but SOC, TN and EC were important in the farmland. In addition, pH had highly significant negative correlations with NO_3_^−^-N and TN (*p* < 0.01). Redundancy analysis (RDA) was further used to elucidate the relation between the bacterial community and soil properties. As shown in Figure 6b, the first two axes explained 29.1% and 9.0% of the total variation in the bacterial community, suggesting the presence of considerable variables that have not been incorporated into the present study. A previous study confirmed that plant species, as compared with irrigation with treated wastewater, had similar magnitude effects on the soil bacterial community [47]. Additionally, treated wastewater irrigation may lead to a substantial enrichment of heavy metals [48], which also significantly affected soil microbial community composition and network interactions [49]. For the entire community composition, the significant effects ranked by R^2^ values in decreasing order were NO_3_^−^-N (0.7198), pH (0.6894), TN (0.5428), AP (0.4931), SOC (0.4739) and EC (0.4051) (all *p* < 0.05). Only NH_4_^+^-N had weak and insignificant effects (R^2^ = 0.0023, *p* = 0.975), which were consistent with the results of the Mantel test, indicating that, among the environmental factors determined, NO_3_^−^-N and pH served as the primary ecological drivers shaping the soil bacterial community (R^2^ > 0.6, *p* < 0.05), while TN, AP, SOC and EC constituted secondary influential factors (0.6 > R^2^ > 0.4, *p* < 0.05). A similar conclusion was reached recently from a study of the long-term impacts of irrigation with municipally treated wastewater on soil properties and microbial communities; soil pH significantly impacted bacterial communities [9]. Moreover, the vertical distribution pattern of soil microbial communities was predominantly shaped by soil TN and pH [50]. These results supported the previous finding that pH was one of the main driving factors accounting for variation in soil bacterial community structure and composition in agroecosystems [51,52].

The correlation between the dominant bacterial genera and the primary drivers can also be seen in Figure 6b. *Nitrospira* bacteria negatively correlated with NO_3_^−^-N and positively correlated with pH. A previous study proved that nitrate with high concentrations could inhibit the growth of *Nitrospira*, and some specific *Nitrospira* species adapted to the highly alkaline environments [53]. *Sphingomonas* displayed a stronger positive correlation with TN than other edaphic factors while demonstrating a negative correlation with pH, suggesting its crucial role in soil N cycling [54] and a low tolerance to alkaline conditions. *Nocardioides* and *Arthrobacter* exhibited positive correlations with NO_3_^−^-N but negative correlations with pH. Therefore, the decrease in their relative abundance in the grassland may be attributed to the increased pH resulting from irrigation.

## 3. Materials and Methods

### 3.1. Experimental Design

The study site is located in Gaotang County, Shandong Province, China (36°51′ N, 116°14′ E). This region has a warm temperate semi-humid continental monsoon climate, with an average annual precipitation of 589 mm and an average temperature of about 13 °C. Since 2017, a test field has been established to cultivate fodder crop *Symphytum officinale* using root segment as explants with a plant spacing of 0.5 m × 1.35 m, and was irrigated with anaerobic digested poultry wastewater under an irrigation amount of 187 mm during the growth season (April to October). The irrigation with TPW continued for six years before the sampling date. The basic properties of TPW are shown in Table 2. The test field was designated for five replications. As the test field planted with *S. officinale* (hereinafter referred to as grassland) was converted from the conventional farmland with a winter wheat–summer corn crop rotation, this study selected adjacent conventional farmlands with the same crop rotation as the control (hereinafter referred to as farmland). For farmland, fertilizer N (400 kg ha^−1^ yr^−1^) was applied in equal portion to both wheat and corn; 25% of the annual total was applied at wheat sowing, 25% at the wheat regreening stage and 50% at the maize tasseling stage. Fertilizer P (60 kg ha^−1^ yr^−1^) and fertilizer K (65 kg ha^−1^ yr^−1^) were applied only in the winter wheat season. Wheat was irrigated three times and corn was irrigated two times through flood irrigation with an irrigation volume of 60 mm each time.

### 3.2. Plant and Soil Sampling

Plant and soil samples were collected in 2023.

*S. officinale* were mowed four times at 5 cm height during the growing season (May, July, August and October). In each mowing, five plants were randomly selected in each plot, and were oven-dried to a constant weight at 70 °C after 15 min of fixation at 105 °C for the measurement of the hay yield, and then the dry samples were ground to determine their N contents.

Five soil profiles in an “S” shape were collected at three depths of 0–20, 20–40 and 40–60 cm using a sample probe in each plot of the grassland, and a composite soil was created for a certain soil depth by mixing all five soil samples at this depth. The soil sampling method for the farmland was identical to that of the grassland. Soil samples were sieved through a 2.0 mm sieve to remove visible impurities, then divided into two sub-samples: the first was used for soil property measurement, the second was stored in –80 °C for microbial analysis.

### 3.3. Soil Chemical Analyses

The soil pH and EC were examined in a 1:2.5 soil/water ratio using a pH electrode and EC meter (Mettler, Giessen, Germany), respectively. TN was determined using the Kjeldahl method. Soil organic matter (SOM) was determined using the potassium dichromate (K_2_Cr_2_O_7_) oxidation–titration method. AP was extracted with sodium bicarbonate and measured using the molybdenum blue method. Soil NH_4_^+^-N and NO_3_^−^-N were determined using a continuous flow analyzer (Auto Analyzer 3, SEAL, Mequon, WI, USA).

### 3.4. Bacterial Community Analysis

Soil DNA was extracted using the E.Z.N.A.^®^ soil DNA Kit (Omega Bio-tek, Norcross, GA, USA) according to the manufacturer’s instructions. The hypervariable region (V3–V4) of the bacterial 16S rRNA gene was amplified with primer pairs 338F and 806R by a PCR thermocycler (BIO-RAD, Hercules, CA, USA). The PCR product was extracted from 2% agarose gel, purified using the PCR Clean-Up Kit (YuHua, Shanghai, China) and quantified using Qubit 4.0 (Thermo Fisher Scientific, Waltham, MA, USA). The purified amplicons were then pooled in equimolar amounts and paired-end sequenced on an Illumina sequencing platform (Illumina, San Diego, CA, USA). The extraction, PCR amplification and sequencing were implemented with technical support from Majorbio Bio-Pharm Technology Co. Ltd. (Shanghai, China). Based on the Majorbio Cloud platform (https://cloud.majorbio.com), bacterial alpha diversity indices (Chao1 richness and Shannon index) were computed, and the differentiation of bacterial community similarity was elucidated through Adonis analysis and principal coordinate analysis (PCoA) based on Bray–Curtis dissimilarity. The linear discriminant analysis (LDA) effect size (LEfSe) was performed to identify the significantly abundant taxa (phylum to genera) of bacteria among the different groups (LDA score > 3, *p* < 0.05). The Mantel test and redundancy analysis (RDA) were performed to examine the relationship between soil physicochemical properties on soil bacterial community structure.

### 3.5. Data Analysis

The experimental data were presented as the mean ± SE. The statistical comparison was made using the SAS 9.4 software. The significance of difference between grassland and farmland was determined with Student’s *t*-test, and that between soil depths was determined with Duncan’s multiple range test. *p* values of less than 0.05 were taken as statistically significant.

## 4. Conclusions

*S. officinale* exhibited strong adaptability to long-term exposure to TPW, and played a predominant role in alleviating N accumulation in soil from TPW irrigation by efficiently absorbing and utilizing N, implying its promising application in the crop–livestock system. Compared with the adjacent conventional farmlands, grassland under long-term TPW irrigation had significantly higher soil pH in all depths and EC in subsoil, while significantly lower levels of NO_3_^−^-N in all depths and TN in topsoil. The composition and structure of the bacterial community in grassland remarkably shifted, and the relative abundance of nitrite-oxidizing bacteria *Nitrospira* and ammonifying bacteria *Flavobacterium* and *Pseudomonas* significantly increased, while those of s-triazine herbicide degrader *Nocardioides* and *Arthrobacter*, as well as *Sphingomonas*, having a low tolerance to saline–alkaline stress, significantly decreased. The determined soil properties explained 38.1% of the variation in bacterial composition, with NO_3_^−^-N and pH exhibiting particularly high importance. Further studies are needed to explicitly address other major contributors like plant species to the shift in bacterial community.

## Figures and Tables

**Table 1:** Yields and N accumulation of *S. officinale*.

The Number of Mowings	Dry Weight t ha^−1^	N Content in Plants g kg^−1^	N Accumulation in Plants kg ha^−1^	N Input by Irrigation with TPW kg ha^−1^	N Removal by Plants %
1	3.08 ± 0.77 ^a^ *	23.08 ± 2.01 ^c^	73.45 ± 13.55 ^b^	514.69	65.95
2	3.75 ± 0.75 ^a^	26.66 ± 2.95 ^c^	100.16 ± 24.39 ^a^		
3	3.20 ± 0.11 ^a^	31.79 ± 3.06 ^b^	101.59 ± 6.76 ^a^		
4	1.65 ± 0.27 ^b^	39.01 ± 3.73 ^a^	64.21 ± 10.70 ^b^		
Total	11.68	/	339.41		

* Different letters in the same column indicate significant differences according to Duncan’s test (*p* < 0.05). All data are expressed as means ± SE.

**Table 2:** Basic properties of TPW.

pH	EC mS·cm^−1^	AP mg·L^−1^	TN g·L^−1^	NH_4_^+^-N mg·L^−1^	NO_3_^−^-N mg·L^−1^	COD mg·L^−1^
7.58 ± 0.02	2.16 ± 0.04	1.93 ± 0.38	0.27 ± 0.08	38.73 ± 10.61	Not detected	390.23 ± 15.17

EC: electrical conductivity; AP: available phosphorus; TN: total nitrogen; COD: chemical oxygen demand. All data are expressed as means ± SE.

## Data Availability

The raw data supporting the conclusions of this article will be made available by the authors on request.
